# Sulforaphane—A Compound with Potential Health Benefits for Disease Prevention and Treatment: Insights from Pharmacological and Toxicological Experimental Studies

**DOI:** 10.3390/antiox13020147

**Published:** 2024-01-25

**Authors:** Katarina Baralić, Jovana Živanović, Đurđica Marić, Dragica Bozic, Lazar Grahovac, Evica Antonijević Miljaković, Marijana Ćurčić, Aleksandra Buha Djordjevic, Zorica Bulat, Biljana Antonijević, Danijela Đukić-Ćosić

**Affiliations:** Department of Toxicology “Akademik Danilo Soldatović”, Faculty of Pharmacy, University of Belgrade, Vojvode Stepe 450, 11221 Belgrade, Serbia; jovana.zivanovic@pharmacy.bg.ac.rs (J.Ž.); djurdjica.maric@pharmacy.bg.ac.rs (Đ.M.); dragica.jorgovanovic@pharmacy.bg.ac.rs (D.B.); lazargrahovac@gmail.com (L.G.); evica.antonijevic@pharmacy.bg.ac.rs (E.A.M.); marijana.curcic@pharmacy.bg.ac.rs (M.Ć.); aleksandra.buha@pharmacy.bg.ac.rs (A.B.D.); zorica.bulat@pharmacy.bg.ac.rs (Z.B.); biljana.antonijevic@pharmacy.bg.ac.rs (B.A.); danijela.djukic.cosic@pharmacy.bg.ac.rs (D.Đ.-Ć.)

**Keywords:** phytochemical, sulforaphane, disease prevention, anticancerogenic effects, mechanisms, toxicity

## Abstract

Sulforaphane (SFN), which is a hydrolysis product from glucoraphanin, a compound found in cruciferous vegetables, has been studied for its potential health benefits, particularly in disease prevention and treatment. SFN has proven to be effective in combating different types of cancer by inhibiting the proliferation of tumors and triggering apoptosis. This dual action has been demonstrated to result in a reduction in tumor size and an enhancement of survival rates in animal models. SFN has also shown antidiabetic and anti-obesity effects, improving glucose tolerance and reducing fat accumulation. SFN’s ability to activate Nrf2, a transcription factor regulating oxidative stress and inflammation in cells, is a primary mechanism behind its anticancerogenic and antidiabetic effects. Its antioxidant, anti-inflammatory, and anti-apoptotic properties are also suggested to provide beneficial effects against neurodegenerative diseases. The potential health benefits of SFN have led to increased interest in its use as a dietary supplement or adjunct to chemotherapy, but there are insufficient data on its efficacy and optimal doses, as well as its safety. This review aims to present and discuss SFN’s potential in treating various diseases, such as cancer, diabetes, cardiovascular diseases, obesity, and neurodegenerative diseases, focusing on its mechanisms of action. It also summarizes studies on the pharmacological and toxicological potential of SFN in in vitro and animal models and explores its protective role against toxic compounds through in vitro and animal studies.

## 1. Introduction

In addition to genetic predisposition, the modern way of life increasingly influences the rising incidence of chronic non-communicable diseases such as diabetes, obesity, cardiovascular, neurodegenerative diseases, cancer, and others [1]. Factors such as a sedentary lifestyle, inadequate nutrition, alcohol consumption, smoking, constant exposure to chemicals from the environment (toxic metals, phthalates, bisphenol A, flame retardants, pesticides, etc.), and many others can contribute to health deterioration and the development of the aforementioned diseases [2,3]. It has been shown that changing lifestyle habits, like quitting smoking, staying at a healthy weight, being physically active, cutting back on alcohol, and eating fruits and vegetables, can lower the risk of disease [4]. Moreover, increased intake of fruits and vegetables can reduce the overall risk of developing cancer and cardiovascular diseases [5]. Accordingly, many plant-derived chemicals, called phytochemicals, benefit human health, prevent disease, and aid in treating some illnesses [6,7]. Chemically, the most important phytochemicals are phenols and polyphenolic compounds, terpenoids, alkaloids, sulfur compounds, etc. [7]. A number of phytochemicals are known for their beneficial health effects, such as berberine [7,8], resveratrol [9], emodin [10], ellagic acid [7], etc. One of the best-known representatives of this group is SFN, chemically 1-isothiocyanato-4-(methylsulfinyl) butane, whose pharmacological and toxicological properties have been extensively studied.

Isothiocyanates are a group of compounds dominantly represented in the cruciferous family (broccoli, cauliflower, kale, cabbage, and others) of vegetables. Epidemiological and clinical studies have shown that increased intake of these vegetables can have a beneficial effect on the body and reduce the chance of developing certain diseases [11]. These compounds are products of the hydrolysis of glucosinolates, secondary metabolites of plants from the *Brassicaceae* family, which are considered to have no biological activity, while the hydrolysis is catalyzed by the enzyme endogenous myrosinase (β-thioglucosidase) or human gastro-intestinal microbiota [12,13,14]. One of the best-known representatives of this group whose pharmacological and toxicological properties are examined is SFN, chemically 1-isothiocyanato-4-(methylsulfinyl) butane. Plants from the *Brassicaceae* family contain glucoraphanin, which, through enzymatic hydrolysis, produces SFN (the aglycone part of this glucosinolate). This process of converting glucoraphanin into SFN is a defense mechanism of the plant and is activated when the plant is exposed to damage in any way (pathogenic organisms and infections, or mechanical damage such as chopping, chewing, etc.). When plant cells are damaged, myrosin cells release myrosinase, which catalyzes the aforementioned reaction. Because of the potential of this interaction taking place when there is no damage to plant cells, myrosinase and glucoraphanin are spatially separated [4,7]). It has been shown that SFN has numerous beneficial effects on the human body. For example, it has been shown to exhibit anti-inflammatory and antioxidant effects. Also, it has been shown that it potentially has anti-tumor, antidiabetic, cardioprotective, and other beneficial health effects [15,16,17].

Having all of this in mind, the aim of this review is to: (i) present and discuss the potential of SFN in treating cancer, but also other diseases such as diabetes mellitus, cardiovascular diseases, obesity, and neurodegenerative diseases, with a focus on the mechanisms through which SFN exerts its effects; (ii) provide a qualitative review of the studies on the pharmacological and toxicological potential of SFN in in vitro and animal models (i.e., mice, rats, and zebrafish); (iii) explore the protective role of SFN against the toxicity of various toxic compounds, including toxic metals, phthalates, and bisphenol A, through in vitro and animal studies; and (iv) address the need to assess the safety of SFN use.

## 2. SFN—General Information

In its liquid state, SFN appears faintly yellow, has a molecular weight of approximately 177.28 g/mol, and possesses the molecular formula C_6_H_11_NOS_2_. SFN typically melts around 74.6 °C, but its melting point can vary between 58.6 °C and 91.2 °C. It exhibits solubility in solvents such as DMSO, methanol, and water-soluble substances [18]. Exposure to elevated oxygen levels, high temperatures, or a basic environment readily leads to the degradation of SFN. There are two forms of sulforaphane: naturally occurring R-SFN and synthetic R,S-SFN. The synthetic form is predominantly utilized for research purposes, in animal studies, where it is applied per os, intraperitoneally or topically [19]. When it enters the body, SFN is easily absorbed and has high bioavailability (around 80%) due to its lipophilicity and structure [14,20,21]. SFN is metabolized via the mercapturic acid pathway. There is an interaction between the reactive electrophilic carbon from the isothiocyanate functional group (–N=C-S) and glutathione (GSH), while this reaction is catalyzed by glutathione S-transferase. The resulting SFN-GSH complex is under the influence of the enzymes c-glutamyltranspeptidase, cysteinylglycinase, and N-acetyltransferase, and the final and main metabolite that is formed is SFN-N-acetylcysteine. SFN has high bioavailability and is quickly metabolized and excreted from the body through urine, and after entering the cells, its accumulation occurs [4,14].

## 3. Mechanisms of SFN-Linked Beneficial Effects

Sulforaphane’s anticancer efficacy has been examined extensively in a variety of malignancies, including cervical, breast, bladder, renal, lung, colon, and prostatic cancers [22]. The isothiocyanate functional group (–NCS) in the SFN molecule is the most critical pharmacophore [23]. The central carbon atom within the –NCS group in the majority of isothiocyanates (ITCs) exhibits a pronounced electrophilic nature, enabling interactions with nucleophiles centered on oxygen, sulfur, or nitrogen. This reactivity leads to the formation of thiocarbamates, dithiocarbamates, or thiourea derivatives, depending on the specific nucleophile involved, and compounds containing this central carbon atom in their structure readily undergo reversible reactions with thiols under physiological conditions [19,24].

Several potential mechanisms of SFN’s anticancerogenic effects have been suggested. SFN effectively inhibits cell cycles in G2/M, but also in the G1/S phase [23]. Induction of apoptosis has also been highlighted as an important mechanism, while SFN has been found to interact with both intrinsic and extrinsic apoptotic pathways [23,25]. It has also been proposed to interfere with cancer initiation by altering metabolic enzymes, leading to the inhibition of carcinogen-activating phase I enzymes (e.g., decreasing CYP1A1 and CYP3A4 activity), and the activation of carcinogen-detoxifying phase II enzymes [20,23]. Furthermore, SFN is recognized for its potent free radical scavenging characteristics, binding numerous oxidants, including superoxide, peroxide, and hydroxyl radicals [20]. Detoxification of electrophiles and oxidants as a result can protect against carcinogens, oxidative stress, and inflammation [26]. It has been demonstrated that SFN regulates balance in the body at the molecular level by activating the transcription factor, nuclear erythroid 2-related factor-2 (Nrf2) [20,27]. This isothiocyanate was found to induce Nrf2 accumulation due to an inhibition of proteasomal degradation of the basic region-leucine zipper (bZIP) protein. SFN can react with protein thiol groups to create thionoacyl adducts, which influences the key Keap1 cysteine residues and prevents Nrf2 polyubiquitination and breakdown, culminating in Nrf2-Keap1-ARE signaling and Nrf2 nucleocytoplasmic redistribution [26]. Nrf2 performs an important anti-inflammatory function in numerous tissues by activating phase II enzymes and, subsequently, by suppressing the nuclear factor-kappa beta (NF-B) signaling pathway [27]. SFN stimulation of Nrf2 results in the production of a number of cytoprotective genes with anticarcinogenic properties. Some of these include NAD(P)H quinone oxidoreductase-1 (NQO1), heme oxygenase 1 (HO-1), catalase, glutamate-cysteine ligase (GCL), glutathione-S-transferase (GST), UDP-glucuronosyltransferase (UGT), epoxide hydrolase, and superoxide dismutase (SOD). Activations of mitogen-activated protein kinase (MAPK), phosphatidylinositol 3-kinase (PI3K), and protein kinase C (PKC) pathways and epigenetic changes are also suggested as potential SFN anticarcinogenic mechanisms [22]. SFN has also been shown to suppress histone deacetylases (HDACs) as well as topoisomerases I and II, all of which play critical roles in DNA replication [25].

In addition to the prevention of carcinogenesis, SFN has also been linked with beneficial properties against cardiovascular system-related disorders, such as hypertension, atherosclerosis, and ischemia–reperfusion (I/R) injury [26]. It has been suggested to have cardiovascular-protective effects through activating the Nrf2 signaling pathway, suppressing inflammatory pathways, and modulating lipid metabolism [28].

This isothiocyanate has also been demonstrated to increase lipolysis and prevent adipocyte differentiation, while this said effect has been connected with decreased expressions of the transcription factors PPARγ and C/EBPα, involved in the regulation of adipocyte differentiation and fat accumulation. Anti-obesogenic SFN effects include the increment of apoptosis, activation of AMPK and fatty acid oxidation pathway, triacylglyceride synthesis pathway activation, increased glucose uptake, and reduced oxidative stress [29]. Additionally, SFN has been found to promote ribosome biogenesis, reduce ROS accumulation, and decrease inflammation in fatty tissue, therefore leading to protection from obesity [30]. SFN’s contribution to lipolysis has also been suggested, through the activation of hormone-sensitive lipase and the browning of white adipocytes. Results suggest that SFN may provoke lipophagy through AMPK–mTOR–ULK1 pathway signaling, resulting in partial lipolysis of adipocytes [31].

Regarding its antidiabetic effects, SFN was found to reduce insulin resistance via the PI3K/Akt and JNK/IKK, AMPK/mTOR pathways, enhance glucose transport via the IRS-1/Akt/GLUT4 and PPAR/GLUT4 pathways, and improve blood glucose levels via the PPAR/GSK/GS pathway [32].

On the other hand, SFN’s effects through the Nrf2 pathway, such as the activation of genes and molecules with antioxidant, anti-inflammatory, and anti-apoptotic properties, have been suggested as the main mechanisms behind SFN’s beneficial effects against neurotoxicity, particularly against neurodegenerative diseases such as Alzheimer’s disease (AD), Parkinson’s disease (PD), and multiple sclerosis (MS) [33].

**Figure 1:**
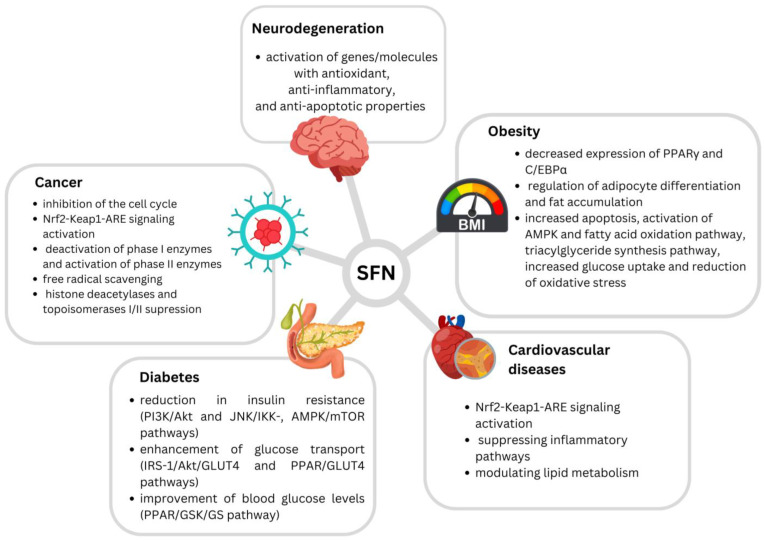
Proposed mechanisms of sulforaphane’s beneficial effects (Houghton et al. (2016) [20]; Kamal et al. (2020) [23]; Mangla et al. (2021) [25]; Bai et al. (2015) [26]; Babu et al. (2017) [28]; Martins et al. (2018) [29]; Çakır et al. (2022) [30]; Wang et al. (2022) [32]; Schepici et al. (2020) [33]).

Proposed mechanisms of SFN’s beneficial effects are presented in Figure 1.

## 4. The Potential of SFN in Disease Prevention and Treatment

### 4.1. Anticancerogenic Effects

Several in vitro studies assessing the beneficial effects of SFN have been conducted, resulting in relevant information regarding the prevention and treatment of diseases such as pancreatic cancer, breast cancer, lymphomas, liver cancer, leukemia, and prostate cancer [34]. For example, SFN was found to restore the loss of gap junction intercellular communication and connexin 43 expression, which were connected with therapy resistance of pancreatic cancer, and thus SFN also restored therapy sensitivity [35]. These antiproliferative effects of SFN on primary effusion lymphoma (PEL) cells were mediated via apoptosis through the activation of caspases. Additionally, in these cells, SFN inhibited the phosphorylation of p38 mitogen-activated protein kinase (p38MAPK) and AKT in PEL cells [36]. Additionally, in vitro studies have indicated that SFN can be regarded as a good supportive therapy to other anti-tumor drugs and chemotherapy in general. For instance, insufficient cellular uptake, deactivation by thiol-containing species, and nonspecific distribution of cisplatin result in its low chemosensitivity, as well as systemic side effects, which can largely constrain the employment of this drug in clinical treatment. Results show that, compared with combinational treatments of cisplatin and SFN, the nanoparticles were more effectively internalized and could significantly reduce GSH content in breast cancer cells, leading to a notable increase in DNA-bound Pt and DNA damage-induced apoptosis [37]. SFN’s effects were also evaluated on imatinib (IM)-resistant leukemia stem cells (LSCs): Combined treatment using IM and SNF sensitized CD34+/CD38-LSCs and induced apoptosis, shown by increased levels of caspase 3, PARP, and Bax, and decreased Bcl-2 expression. Mechanistically, IM–SFN combined treatment resensitized LSCs by inducing the production of intracellular reactive oxygen species (ROS). Importantly, β-catenin-silenced LSCs exhibited reduced glutathione S-transferase pi 1 (GSTP1) expression and intracellular GSH levels, which led to increased sensitivity toward IM and SFN. It was demonstrated that IM–SNF combined treatment effectively eliminated CD34+/CD38-LSCs [38]. SFN in combination with other phytochemicals has also shown beneficial effects in certain cell lines. SFN and capsaicin, a chemical compound first isolated from chili peppers, decreased the number of nuclear androgen receptors, prostate-specific antigen and Bcl-XL levels, and cell proliferation induced by androgen and Tip60 in LNCaP cells—a cell line derived from a metastatic lymph node lesion of a human androgen receptor positive for prostate cancer. These bioactive compounds prevented increases in glycolysis, hexokinase, and pyruvate kinase activity, and reduced HIF-1α stabilization induced by androgen and Tip60 in LNCaP cells. The protective role of sulforaphane and capsaicin in prostate cancer may rely on mechanisms involving the inhibition of Tip60, AR, and HIF-1α effects [39].

Additionally, numerous in vivo animal studies have been conducted indicating the anti-tumor potential of SFN. It can act on tumors of various organs such as the breast, colon, esophagus, skin, etc., leading to a reduction in tumor size, mostly by inhibiting proliferation and inducing apoptosis of malignant cells [40]. In an in vivo study on rats by Castro et al. (2019), SFN showed significant efficacy against triple-negative breast cancer by inhibiting the proliferation and formation of stem-like cancer cells responsible for resistance to chemotherapy and radiotherapy due to its high capacity for self-renewal. SFN resulted in a significant reduction in the number and size of these cells. It was also shown that rats treated with SFN prior to tumor induction were more efficient [41]. Tsubura et al. also demonstrated the efficacy of SFN in treating mammary tumors in female mice by achieving dose-dependent suppression of proliferation and induction of apoptosis of KLP-1 tumor cells, and reducing metastasis to lymph nodes [42]. Lu et al. demonstrated the anticancer efficacy of SFN in esophageal squamous cell tumors, where it inhibited proliferation and induced apoptosis by activating caspase-mediated signaling pathways, but also autophagy, through the activation of Nrf2. In this study, however, it was shown that SFN at low doses (5 mg/kg b.w.) induces tumor-protective autophagy, which helps cancer cells cope with metabolic stress and promotes tumor cell survival. Due to this, at this dose level, SFN could not achieve the expected anticancer effect [43]. However, Nrf2 activation does not exclusively contribute to tumor-protective autophagy, as shown in the previous study, but may also contribute to the anti-tumor activity of SFN, as shown by Byun et al. who investigated whether the same dose of SFN (5 mg/kg b.w.) could be used against tumors. Low SFN doses led to a dose-dependent reduction in the size of colon tumors, through the increase in the activity of proteins involved in the cell cycle. As a part of this investigation, the same authors also performed an in vitro study, in which it was shown that SFN led to a decrease in the level of cellular glutathione and consequently an increased production of ROS in tumor cells, whereby the cancer cells were more sensitive compared to the healthy cells. Also, by activating Nrf2 signaling, SFN increased the level of ROS in tumor cells. Here, the stress-activated kinase p38, activated by increased ROS generation and SFN-induced phosphorylation, activated other signaling proteins, resulting in cell cycle arrest and apoptosis [44]. SFN was also shown to act on pancreatic tumor cells by inhibiting their growth and, consequently, reducing tumor progression and increasing the survival rate, which was shown by Chen et al., 2019, when treating BALB/c mice (transgenic pancreatic cancer mice) with SFN for 120 days [45]. In lung tumors, SFN prevented the epithelial–mesenchymal transition, which has a key role in all stages of lung cancer development and progression, leading to a decrease in the migratory and invasive capacity of lung cancer cells through increasing the activity of ERK5, extracellular signal-regulated kinase 5 [46]. However, even if the anti-tumor activity of SFN is known and proven many times, in the study conducted by Rai et al., 2020, the independent application of SFN did not give the expected results. But, the combination of paclitaxel and SFN led to a significant reduction in tumor size, without exhibiting toxic effects, which indicates the potential of SFN to be administered as an adjunct to chemotherapeutics to reduce side effects [47]. In addition to the studies conducted on mice in which the anti-tumor property of SFN was examined, numerous studies have been conducted on zebrafish due to their genetic homology and the similarity of their molecular pathways to humans, as well as for simpler and faster execution of experiments. In this model, the effectiveness of SFN in reducing the growth of glioblastomas, breast cancer, and cervical tumors was demonstrated [48]. Also, it has been demonstrated that SFN can affect melanogenesis by increasing melanin biosynthesis through its effect on the MITF (microphthalmia-associated transcription factor) PCKB1 and protein tyrosinase, thereby inhibiting the proliferation and migration of melanoma cells and reducing their metastasis [49].

A summary of animal studies demonstrating the anticancerogenic effects of SFN is presented in Table 1.

### 4.2. Antidiabetic/Anti-Obesogenic Effects

Furthermore, SFN has shown positive effects by effectively increasing glucose uptake and improving insulin signaling in palmitic acid (PA)-induced HepG2 cells. SFN has also led to increased expression of antioxidant genes downstream of Nrf2 and decreased accumulation of lipid peroxides MDA and 4-HNE. In PA-induced HepG2 cells and flies, the alleviation of insulin resistance by SFN was diminished by the GPx4 inhibitor. Taken together, SFN ameliorated HFD-induced insulin resistance by activating the AMPK–Nrf2–GPx4 pathway, providing new insights into SFN as a therapeutic compound for the alleviation of insulin resistance [51]. Due to the increasing need to utilize substances of plant origin and the biological activity of SFN in the prevention and treatment of diseases, numerous studies have been conducted on their importance in the treatment of diabetes. Axelsson et al. (2017) demonstrated the antidiabetic activity of SFN in mice and rats and subsequently confirmed it in a human study. Treatment of these mice and rats with different doses of SFN resulted in a decrease in glucose production due to the translocation of Nrf2 and the reduction in enzymes important for gluconeogenesis. In addition, the effect of SFN was compared with the effect of metformin, the first-line agent for the treatment of diabetes mellitus type II, showing no significant difference, but demonstrating that they achieve their effect through a different mechanism [52]. In addition to the potential of SFN in the treatment of diabetes, its contribution to the prevention of the development of macrovascular complications in diabetes is also well known. The protective effect on the development of retinopathy, nephropathy, and cardiovascular disease is evident in the activation of Nrf2, which contributes to the increase in the antioxidant capacity of somatic cells [53,54,55]. Li et al. (2019) showed that increased activity of the Nrf2 pathway leads to increased gene expression for heme oxygenase 1 (HO-1) and NAD (P)H oxidoreductase (NQO1), and the induction of gene expression for antioxidant enzymes (GSH, SOD, CAT). In addition, SFN decreases the level of inflammatory cytokines (TNFalpha, IL1beta, IL6) and the expression of inflammatory components (NLRP3, cleaved caspase 1 p20, IL1beta p17, and ASC), which contributes to the prevention of diabetic retinopathy [53]. In addition, activation of the Nrf2 pathway induces apoptosis and ferroptosis, preventing the progression of diabetic cardiomyopathy, as shown in a study by Wang et al. (2022). Ferroptosis was shown to be critical in the development of cardiomyopathy even 6 months after the development of diabetes in mice, whereas apoptosis was important for the early stages of cardiomyopathy development [54]. Khaleel et al. (2019) demonstrated that SFN has a protective effect on the kidneys and prevents the development of nephropathy through the activation of Nrf2 and HO-1, and suppression of the expression of IL6 and caspase 3 [55].

Type II diabetes is usually associated with obesity, which together present a growing global problem causing numerous health, economic, and social problems in the world [56]. Because SFN is able to affect lipid metabolism, animal studies have investigated whether it can help to reduce obesity. Liu et al. (2021) showed that SFN suppressed body weight gain and reduced adipocyte size and the accumulation of lipids in obese female mice, through its effect on the expression of genes involved in lipid metabolism and mitochondrial oxidative stress. This suggests the possibility of SFN as a potential anti-obesity drug [57]. SFN may also reduce oxidative and inflammatory damage caused by obesity-related glomerulopathy by activating Nrf2 and promoting autophagy, as shown by Lu et al. (2020) in a study of mice [58].

A summary of animal studies demonstrating the antidiabetic/anti-obesogenic effects of SFN is presented in Table 2.

### 4.3. Cardiovascular-Protective Effects

The use of phytochemicals such as SFN could play an important role in the prevention of cardiovascular diseases, as numerous in vitro and in vivo studies have demonstrated. Poletto Bonetto et al. (2022) showed that SFN exerts a cardioprotective effect by reducing the expression of the ryanodine receptor (Ryr), leading to modulation of myocardial contraction and Ca handling in rats with cardiac ischemia [59]. Jayakumar et al. (2013) indicated the potential of SFN for use as a dietary supplement in the prophylaxis of acute pulmonary thromboembolism. SFN acted protectively by activating adenylate cyclase, resulting in an increase in cAMP levels and subsequent inhibition of signaling pathways, which, in turn, resulted in an inhibition of platelet aggregation [60]. In a study conducted by Zhang et al. (2022), SFN increased cardiac function and cardiomyocyte survival in mice with induced cardiac ischemia. This cardioprotective effect was achieved by inhibiting the expression of the CAMK2D gene (which encodes the structure of the protein CaMKIIδ, whose reduced activity protects the heart from ischemic damage) and inducing gene expression for the CaMKIIN2 protein, which inhibits CaMKIIδ [61]. Bai et al. (2017) indicated that SFN may have a protective effect on cardiac function through activation of Nrf2, inhibiting myocardial hypertrophy and fibrosis, inflammation, and oxidative stress [62].

A summary of animal studies demonstrating the cardiovascular health benefits of SFN is presented in Table 3.

### 4.4. Neuroprotective Effects

Several in vitro studies have focused on neurodegenerative diseases and the potential beneficial effects of SFN. In one in vitro study on the primary cortical cells of rats, it was observed that SFN (0.1 μM) improved the viability as well as preserved the dendritic length of neurons, which was reduced following Aβ oligomer incubation. Therefore, the results of such study demonstrate that SFN can be useful to counteract the Aβ aggregation in Alzheimer’s disease [63]. In order to investigate the correlation between Nrf2 and Alzheimer’s disease, Bahn et al. showed the effects of SFN in vitro and in vivo. The authors demonstrated that SFN (1 μM), in SH-SY5Y cells, led to the overexpression of Nrf2 and reduced the transcription level of BACE1 and BACE1-AS, involved in amyloidogenesis processes [64]. In the case of Parkinson’s disease, SFN exerted this protective effect, probably through the upregulation of the Nrf2–ARE pathway, as shown by the reversion in the reduction in HO-1 and NQO-1 antioxidant enzyme expression, which was reduced following MPP+. Consequently, SFN also reduced oxidative stress and prevented cell damage [65]. On the other hand, numerous studies have used in vivo models to investigate how SFN affects cognition in nervous system diseases to determine whether it can be used for prophylaxis and therapy of neurodegenerative diseases, such as Alzheimer’s disease, Parkinson’s disease, and multiple sclerosis. For example, Zhang et al. (2015) pointed out the possibility of using SFN in therapy for Alzheimer’s disease. In mice with lesions of this disease, SFN achieved neuroprotective effects by protecting the brain from amyloid β (Aβ)-deposits responsible for the degeneration of neurons and synapses, restoring endogenous antioxidants in the brain, and regulating the activity of glutathione peroxidase (GPX), whose activity is reduced in mice with Alzheimer’s disease. This study also demonstrated the possible anxiolytic effect of SFN, as it affected reduced locomotor activity in an open-air test of diseased mice [66]. The possibility of using SFN in the prophylaxis of Alzheimer’s disease was also presented in the study by Lee et al. (2018): Regular oral administration of this substance at a daily dose of 10 to 50 mg/kg prevented the memory impairment characteristic of Alzheimer’s disease and reduced the levels of tau, phosphorylated tau, and Aβ-protein, the main pathophysiological factors in this disease. SFN achieved these effects by affecting their production and clearance by increasing the activity of heat shock protein 70, HSP70, and the C-terminus of HSP70-interacting proteins [67]. The beneficial effects of SFN on Alzheimer’s disease were also demonstrated by Hou et al. (2018), who indicated that SFN improved cognition in Alzheimer’s disease and wild-type mice and reduced their level of Aβ-oligomers, which likely led to their improved cognition. These authors also pointed the importance of the timing of initiating SFN therapy. According to their findings, SFN is most effective against neurodegeneration when nerve function is the least damaged, that is, at the onset of abnormal formation of Aβ-oligomers, which is around the sixth month of life in rats. This assumption was also confirmed in an in vitro assay performed as a part of the same study [63]. Pu et al. (2018) and Wang et al. (2020) showed that SFN has a positive effect on cognition and memory and consequently prevents the development of Alzheimer’s disease. Pu et al. (2018) showed that SFN can prevent cognitive decline resulting from diabetes by activating Nrf2, leading to increased antioxidant protection and decreased Aβ protein and tau phosphorylation in the hippocampus [68]. In an experiment by Wang et al. (2020), SFN also reduced depressive-like behavior in rats through effects on serotonin metabolism and transport. This reduction in cognitive decline is a consequence of its effect on inflammatory cytokines and enzymes of antioxidant protection, preventing the memory impairment characteristic of dementia [69]. Morroni et al. (2013), Jazwa et al. (2011), and Zhou et al. (2016) conducted studies in mice investigating the possibility of using SFN in the therapy of Parkinson’s disease. In these studies, SFN had a neuroprotective effect and prevented the degeneration of dopaminergic neurons, resulting in a significant improvement in impaired motor function, coordination, and balance. This effect was achieved by increasing the antioxidant potential of substantia nigra and inhibiting apoptosis [70,71,72]. Inhibition of apoptosis was achieved by decreasing DNA fragmentation and caspase-3 activity, which decreased the degeneration of substantia nigra dopaminergic neurons [70]. In these studies, SFN was suggested to enhance brain antioxidant protection by activating Nrf2 and subsequently the antioxidant enzymes HO-1 and NKO1, and by affecting glutathione metabolism [70,71]. In their study, Zhou et al. (2016) also emphasized the importance of the influence of SFN on autophagy. At a dose of 50 mg/kg, SFN did not activate autophagy on its own. However, it effectively inhibited autophagy, as evidenced by increased expression of the autophagy biomarker, microtubule-associated protein light chain 3 (LC3), in a cell line exposed to SFN, as confirmed through in vitro experiments [71]. Li et al. (2013) conducted studies in mice with autoimmune encephalomyelitis to investigate the possibility of using SFN as an adjunctive therapy for multiple sclerosis. Since multiple sclerosis is characterized by immune-mediated demyelination of neurons in the brain, spinal cord, and optic nerve, SFN prevented neuronal degeneration due to its antioxidant and anti-inflammatory effects. Its antioxidant effect is achieved through the activation of Nrf2 and the consequent increased synthesis and activity of antioxidant enzymes. SFN reduced the number of antigen-specific Th17 cells, critical for the development of autoimmune encephalopathy. Furthermore, it enhanced the integrity of the blood–brain barrier by suppressing oxidative stress through diminishing the expression of MMP-9 (matrix metalloproteinase-9), the tissue inhibitor of metalloproteinase. As a result, it safeguarded the levels of crucial proteins such as claudin-5 and occludin, which play a vital role in preserving the integrity of the blood–brain barrier [73]. In view of these animal studies which have demonstrated the beneficial effects of SFN in the prophylaxis and therapy of various diseases, its use as a neuroprotective agent should be considered as an adjunct to the regular therapy of these diseases. A summary of animal studies demonstrating the neuroprotective effects of SFN is presented in Table 4.

## 5. SFN’s Protective Effects against Toxic Substances

The protective effects of SFN against various toxic substances have been shown in several studies using in vitro and animal models (Table 5). For example, this substance ameliorated the toxicity of bisphenol A (BPA), as well as toxic metal(oid)s such as aluminum (Al), arsenic (As), cadmium (Cd), and chromium (Cr). Overall, it has been found to mitigate oxidative stress, lipid peroxidation, DNA damage, and apoptosis, as well as improving antioxidant status, hormone levels, and tissue changes caused by toxic substances. As seen in the Table 5, in the majority of the conducted studies, and in line with its previously addressed mechanisms of action, SFN was found to activate the PI3K/Akt-mediated Nrf2 signaling pathway [75,76,77,78,79], leading to the upregulation of phase II antioxidant enzymes, such as NAD(P)H quinone dehydrogenase 1 (NQO1), heme oxygenase-1 (HO-1), and gamma-glutamylcysteine synthetase (γ-GCS), which was later connected with reductions in oxidative stress [75,77,80] and the inhibition of inflammatory factors, such as tumor necrosis factor alpha (TNF-α), interleukin-6 (IL-6), and interleukin-1 beta (IL-1β) [76]. Through these mechanisms, SFN protected against hepatic [76,81], renal [78], cardiac [80], respiratory [77], and reproductive [75] toxicities caused by the aforementioned environmental chemicals. All of these findings suggest that SFN may have therapeutic potential for mitigating the toxic effects of various substances in humans. However, further studies are needed to confirm the effectiveness and safety of SFN for this purpose.

## 6. Toxic Effects

Assessing the safety of SFN is important due to its widespread use as a dietary supplement and potential therapeutic agent. While SFN has been found to have various health benefits, including all of the aforementioned beneficial properties, there is also evidence to suggest that it can induce adverse effects. Our research group has used SFN as an example to create an adverse outcome pathway (AOP) linked with potential SFN toxic effects. In total, 11 SFN-related genes associated with chromosomal damage and 146 SFN-related genes linked to skin diseases were observed, respectively, while SFN-triggered protein–protein interactions involved 490 and 1986 proteins associated with chromosomal damage and skin diseases, respectively. SFN was shown to have the ability to cause cell cycle disruption, apoptosis, and immune system activation, whereas chromosomal damage and/or skin illnesses such as dermatitis or psoriasis appeared to be its negative effects [83]. Furthermore, in another investigation, we proposed that SFN might also promote the overexpression of some genes (TIMP1, AURKA, CEP55) while suppressing others (CRYAB, PLCE1, and MMP28), thus leading to the advancement of colorectal cancer. SFN increased RUNX2 transcriptional activation, AURKA stimulation through TPX2, and IL-10 signaling, while inhibiting the process of white and brown adipocyte differentiation, an underlying mechanism whose inactivation might result in obesity, according to our pathway enrichment study. Increases in the expression of these genes have been found to be potentially connected with tumor progression and aggressiveness, while their promotion could also contribute to atherosclerosis and renal fibrosis in chronic kidney disease, respectively [13]. Another in silico analysis conducted by our research group revealed the possibility of SFN hepatotoxicity and dermal adverse effects, but also for Ames toxicity (mutagenicity), rat oral acute toxicity, carcinogenicity, eye corrosion, eye irritation, and respiratory toxicity [84]. However, it is important to note that the evidence regarding this potential SFN toxicity is mostly based on in silico studies. While these studies provide valuable insights into the mechanisms underlying the effects of SFN, their findings are limited and need to be validated through further research and experimental validation. One notable animal study by Socala et al. (2017) found that SFN’s median lethal dose (LD50) in mice was approximately 212.67 mg/kg, with a TD50 of about 191.58 mg/kg. Additionally, when testing the highest SFN dose (200 mg/kg), they observed a significant decrease in thresholds for initiating myoclonic twitch, generalized clonic seizure, and 6 Hz-induced psychomotor seizure [85]. Therefore, it might be concluded that for long-term use of SFN or broccoli-based approaches, it is crucial to carefully consider factors such as dosage, timing, and duration. The impact of SFN on cancer development was also examined, with emphasis placed on the timing of its applications and study design as a crucial factor influencing its effects. Tao et al. (2018) explored the effects of SFN on lung cancer development using two distinct mouse models—one involving the induction of lung cancer through chemicals and the other through genetic manipulation in an LSL-K-rasG12D/+ mouse model. They gave SFN at a dose of 12.5 mg/kg (75.5 µmol/kg) before and after tumor initiation. In the chemical model, SFN before tumor induction reduced tumor numbers, but post treatment, it slightly increased tumor growth. In the genetic model, pre-treatment with SFN had no effect on tumor numbers, but post treatment, it raised both tumor numbers and size [86]. On the contrary, Kombairaju et al. (2012) found that extended inhalation of SFN (0.5 mg, 5 days a week for 3 months) did not worsen tumorigenesis in an identical LSL-K-rasG12D/+ mouse model [87]. While chemically synthetized D,L-sulforaphane is solely available for research purposes [19], glucoraphanin is commercially available as a supplement. On the other hand, broccoli sprouts are a leading natural reservoir of glucoraphanin, which acts as a precursor to SFN. Over the last decade, clinical trials demonstrating SFN’s chemoprotective effects predominantly utilized broccoli sprouts as a dietary source of SFN. In these trials, fresh sprouts with active myrosinase were given doses of around 100 µmol, whereas heat-processed sprouts, lacking this enzyme, required higher doses (400 to 800 µmol) due to reduced SFN bioavailability. It is important to note that caution is advised with glucoraphanin supplements, yet a daily intake of approximately 100 µmol of glucosinolates is generally considered safe [88]. However, in the context of regular dietary consumption, reaching “toxic” levels of SFN would be highly improbable. It is also essential to note that while some studies indicate potential toxicity, these are primarily preclinical and necessitate further toxicological investigations to fully understand the compound’s effects. However, reputable authorities, such as the American Institute for Cancer Research (AICR) and initiatives like the “5-a-day” program, consistently advocate for the inclusion of cruciferous vegetables in a balanced and healthy diet (AICR, 5-a-day) [89,90].

## 7. Conclusions and Future Perspectives

SFN has been extensively studied for its beneficial effects. Its anti-cancer properties have been demonstrated in various types of cancer, such as breast, bladder, renal, lung, colon, and prostate cancers, by inhibiting the cell cycle, inducing apoptosis, and regulating metabolic enzymes. SFN’s ability to scavenge free radicals, detoxify electrophiles and oxidants, and activate the transcription factor Nrf2 has also been found to be beneficial against carcinogens, oxidative stress, and inflammation. This compound has also been linked to the prevention and treatment of cardiovascular diseases, diabetes, and obesity. Its benefits on the cardiovascular system are associated with activating the Nrf2 signaling pathway, suppressing inflammatory pathways, and modulating lipid metabolism. SFN has been shown to reduce insulin resistance, enhance glucose transport, and improve blood glucose levels via different pathways. Moreover, SFN is suggested as a potential treatment for neurodegenerative diseases such as Alzheimer’s disease, Parkinson’s disease, and multiple sclerosis by activating genes and molecules with antioxidant, anti-inflammatory, and anti-apoptotic properties through the Nrf2 pathway. Furthermore, it has been shown to have a protective effect against toxic substances by stimulating the body’s natural detoxification pathways, but also through a direct antioxidant effect (scavenging free radicals and reactive oxygen species, and protecting against oxidative damage to cells and tissues caused by exposure to toxic substances). 

In conclusion, SFN is a promising compound with numerous benefits, making it a potential preventive and therapeutic agent in various diseases. The mechanisms underlying its effects are complex, involving multiple pathways and targets at the molecular level. However, further research is needed to explore the full potential of SFN’s effects and its optimal doses. Furthermore, the safety of SFN must be assessed due to its potential negative effects, including chromosomal damage, skin diseases, tumor progression, and overall toxicity, as suggested by in silico studies. According to the results from the limited animal research, it can be concluded that for long-term use of SFN, it is crucial to carefully consider factors like dosage, timing, and duration. However, more research, including in vitro and in vivo studies, is needed to confirm the potential adverse effects of SFN and to establish safe levels of SFN exposure.

## Figures and Tables

**Table 1:** Anticancerogenic effects of sulforaphane: evidence from animal studies.

Animal Species	Treatment Doses	Duration	Results	References
Transgenic zebrafish Tg	3 µmol phosphate derivative of sulforaphane	48 h	Reduced growth of glioblastomas, breast cancer, and cervical cancer	Rudzinska-Radecka et al. (2021) [48]
Transgenic zebrafish Tg	30 and 50 µmol		Inhibited proliferation Inhibited migration of melanoma cells Reduced metastasis	Eom et al. (2022) [49]
Balb/C nude mice	50 mg/kg ip	5 weeks	Inhibited the growth of breast tumors	Castro et al. (2019) [41]
Athymic BALB/c mice transplanted with KPL-1 cells	25 and 50 mg/kg ip	26 days, 5 times per week	Decreased breast tumor growth	Tsubura et al. (2011) [42]
Nude mice	1 and 5 mg/kg/day ip	13 days	Suppressed colon tumor growth Decreased tumor size	Byun et al. (2016) [44]
BALB/c mice inoculated with ECa109 cells	5 mg/kg, ip	2 weeks, every other day	Decreased esophagus tumor size	Lu et al. (2021) [43]
BALB/c nude mice (transgenic pancreatic cancer mice)	50 mg/kg, ip	120 days, every other day	Reduced pancreatic tumor volume and weight	Chen et al. (2018) [50]
SCID mice inoculated with Ishikawa cells	50 mg/kg ip	30 days	Reduced endometrial tumor volume	Rai et al. (2020) [47]
BALB/c nude female mice inoculated with H1299	25 and 50 mg/kg ip	21 days, every 3 days	Reduced lung tumor weight and volume Inhibited cell migration and invasion	Chen et al. (2019) [46]

**Table 2:** Antidiabetic/anti-obesogenic effects of sulforaphane: evidence from animal studies.

Effects	Animal Species	Treatment Doses	Duration	Results	References
Antidiabetic/anti-obesogenic effects	C57BL/6 mice	10 mg/kg ip	30 days	Decreased adipogenesis Caused browning of white adipocytes	Liu et al. (2021) [57]
	Nrf2−/− (knockout) mice with a C57BL6/J genetic background Wild-type mice	10 mg/kg ip	2 months, every other day	Reduced oxidative and inflammatory damage induced by obesity-related glomerulopathy	Lu et al. (2020) [58]
	Wistar rats C57BL/6J BomTAC mice C57BL/6j mice	2.5 mg/kg 5 mg/kg 10 mg/kg 10 mg/kg ip	15 weeks; 3 times per week; 14 days; 9–12 days; 4 weeks	Reduced glucose production Improved glucose tolerance	Axelsson et al. (2017) [52]
	AMPKalfa2 knockout mice Wild-type C57BL/6J mice	0.5 mg/kg sc	3 months, 5 times per week	Prevented the development of diabetic cardiomyopathy	Wang et al. (2022) [32]
	Wistar rats	3 mg/kg ip	Single dose	Protective effect on the development of diabetic nephropathy	Khaleel et al. (2019) [55]
	Male Sprague Dawley rats	1 mg/kg 0.5 mg/kg ip	12 weeks	Protective effect against diabetes retinopathy	Li et al. (2019) [53]

**Table 3:** Cardiovascular health benefits of sulforaphane: evidence from animal studies.

Effects	Animal Species	Treatment Doses	Duration	Results	References
Cardiovascular health benefits	ICR strain mice	0.125–0.5 mg/kg iv	Single dose	Inhibited platelet aggregation	Jayakumar et al. (2013) [60]
	C57BL/6N mice	50 µg/kg ip	3 days	Increased cardiac function Increased cardiomyocyte survival	Zhang et al. (2022) [61]
	MI model rats	5 mg/kg ip	25 days	Cardioprotective effects from ischemia	Poletto Bonetto et al. (2022) [59]
	Sprague Dawley rats	0.5 mg/kg sc	6 weeks	Prevented fibrosis and cardiac hypertrophy caused by doxorubicin	Bai et al. (2017) [62]

**Table 4:** Neuroprotective effects of sulforaphane: evidence from animal studies.

Effects	Animal Species	Treatment Doses	Duration	Results	References
Neuroprotective effects	C57BL/6 mice	25 mg/kg oral	80 days	Protected the brain from amyloid β (Aβ) deposits	Zhang et al. (2015) [66]
	3 × Tg-AD mice	10 or 50 mg/kg gavage	8 weeks, 6 days per week	Improved memory and learning deficits	Lee et al. (2018) [67]
	Type II diabetes mellitus transgenic mice	1 mg/kg ip	28 days	Prevented cognitive deficit Prevented the development of Alzheimer’s disease as a result of diabetes	Pu et al. (2018) [68]
	PS1V97L transgenic mice	5 mg/kg ip	4 months	Improved cognitive deficits Inhibited Aβ aggregation Inhibited tau hyperphosphorylation Reduced oxidative stress and neuroinflammation	Hou et al. (2018) [63]
	C57Bl/6 mice	5 mg/kg ip	4 weeks, 2 times per week	Prevented motor deficits Prevented loss of dopaminergic neurons	Morroni et al. (2013) [70]
	C57BL/6 mice	50 mg/kg ip	60 days, every other day	Prevented motor deficits Prevented loss of dopaminergic neurons Induced inhibited autophagy	Zhou et al. (2016) [71]
	Wild-type mice Nrf2-KO mice	50 mg/kg ip		Prevented motor deficits Prevented loss of dopaminergic neurons	Jazwa et al. (2011) [72]
	EAE C57Bl/6 mice	50 mg/kg ip	14 days	Improved behavioral deficits	Yoo et al. (2019) [74]
	C57BL/6 mice	50 mg/kg ip	22 days	Reduced oxidative stress Inhibited inflammation	Li et al. (2013) [73]
	Sprague Dawley male rats	5 mg/kg ip	7 days	Improved memory Improved depressive behaviors	Wang et al. (2020) [69]

**Table 5:** Protective effects of sulforaphane against toxic substances: evidence from in vitro and animal studies.

Species	Toxic Substance	Sulforaphane (SFN)	Results	References
human hepatocyte cell line (LO2) and C57/BL6J mice	bisphenol A (BPA): 100, 1000 nM for 24 h	SFN (0.25 μM, 0.5 μM) for 24 h	Ameliorated BPA-altered hepatic lipid metabolism Ameliorated ER stress-related markers	Hong et al., 2023 [81]
mouse Leydig (TM3) cells	Cadmium (Cd): 10 μmol/L for 24 h	SFN (2.5, 5, 10 μmol/L) for 24 h	Reduced the release of lactate dehydrogenase Caused changes in testosterone concentration Increased levels of antioxidant enzymes Inhibited the production of malondialdehyde or reactive oxygen species Protective effect against Cd-induced oxidative stress	Yang et al., 2019 [75]
mouse HepG2 cells	in vitro: cadmium chloride (CdCl_2_): 20 μM for 24 h; in vivo: cadmium chloride (CdCl_2_): 10 mg/kg b.w/day, per os, 4 weeks	in vitro: SFN (0–80 μM) for 24 h; in vivo: SFN (0.5, 1, 2 mg/kg b.w/day), per os, 6 weeks	Antioxidant effects—scavenged free radicals Antioxidant effects—improved redox homeostasis Anti-inflammatory effects—attenuated the expressions of the liver inflammatory factors	He et al., 2021 [76]
mouse alveolar type II epithelial cell line (MLE-12)	in vitro: potassium dichromate (K_2_Cr_2_O_7_): 1 mg/mL for 24 h after SFN	in vitro: pretreating with 0.1 mM SFN for 30 min	Increased the expression of Nrf2 phase II detoxification enzymes Prevented Cr-induced lung toxicity in rats	Lv et al., 2020 [77]
35-day Cr-induced pulmonary toxicity model	in vivo: potassium dichromate (K_2_Cr_2_O_7_): 6, 4, and 2 mg/kg b.w/day, per os, 35 days	in vivo: SFN (4 mg/kg b.w/day), subcutaneous injection	Prevented Cr-induced oxidative stress Prevented histopathological lesions, inflammation, apoptosis, and changes in protein kinase B (Akt) and glycogen synthase kinase 3 beta (GSK-3b) levels	
rats	potassium dichromate (K_2_Cr_2_O_7_): 4 mg/kg b.w/day, intraperitoneal injection, 4 weeks	SFN (4 mg/kg b.w/day), subcutaneous injection 1 h after K_2_Cr_2_O_7_ treatment, 4 weeks	Alleviated hematological variations, oxidative stress, heart dysfunction and structure disorder, and cardiomyocyte apoptosis induced by K_2_Cr_2_O_7_ Ameliorated Cr(VI)-induced cardiotoxicity via activation of the Sesn2/AMPK/Nrf2 signaling pathway	Yang et al., 2020 [80]
rats	sodium arsenite (NaAsO_2_): 5 mg/kg b.w/day, per os, 28 days	SFN (80 mg/kg b.w/day), per os, 28 days	Antioxidant effects—attenuated renal reactive oxygen species, 8-hydroxy-2′-deoxyguanosine, lipid peroxidation, and DNA damage Increased phase II antioxidants Protection against Ar-induced nephrotoxicity.	Thangapandiyan et al., 2019 [78]
rats	sodium arsenite (NaAsO_2_) (5 mg/kg b.w/day), oral, 4 weeks	SFN (20, 40, 80 mg/kg b.w/day), oral, 4 weeks (administered 90 min before As)	Protective effect for arsenic-induced oxidative stress Prevented arsenic-induced liver toxicity	Thangapandiyana et al., 2019 [79]
rats	aluminum chloride (AlCl_3_) (100 mg/kg b.w/day), per os	SFN (100 mg/kg b.w), per os	Improved motility, viability, and sperm count, hormone levels and antioxidant status. Reduced tissue changes caused by As (shrinkage of seminiferous tubules, spermatogenesis disruption, and empty lumen).	Ogunlade et al., 2020 [82]
